# Handgrip-Ring Structure Sensing Probe Assisted Multiple Signal Amplification Strategy for Sensitive and Label-Free Single-Stranded Nucleic Acid Analysis

**DOI:** 10.1155/2024/6832856

**Published:** 2024-10-18

**Authors:** Ying Ren, Yu He, Ping Li

**Affiliations:** Department of Pathology, Affiliated People's Hospital of Ningbo University, Ningbo, Zhejiang 315040, China

**Keywords:** exonuclease, handgrip-ring sensing probe, miRNA, rolling circle amplification, ssDNA

## Abstract

Precise and efficient identification of single-stranded nucleic acids is crucial for both pathological research and early diagnosis of diseases, such as cancers. Therefore, we have devised a novel biosensor that utilizes an elegantly designed handgrip-ring structure sensing probe to enhance the detection sensitivity and reduce background signals. The handgrip-ring structure sensing probe combines ring padlock-based target recognition and hairpin structure probe-based signal amplification. The target sequences form a binding interaction with the ring padlock in the sensing probe, leading to the elongation of the single-stranded chain with the assistance of polymerase. This elongation step results in the release of the hairpin probe, triggering a signal amplification process. This design significantly minimized the potential discrepancies that may occur during the signal amplification process, hence bestowing the approach with a low level of background signals. By utilizing this innovative design, the current biosensor demonstrates a remarkable ability to detect miRNA with a limit as low as 376 aM and single-stranded DNA sequences with a limit as low as 45.3 aM. In addition, it possesses exceptional discrimination capabilities. The efficacy of this approach in diagnosing targets was also effectively proved by the rational redesign of the ring padlock.

## 1. Introduction

The rapid and precise identification of single-stranded nucleic acids (e.g., microRNA and miRNA [1–3]; p53 gene [4, 5]) plays a key role in pathological investigation and early diagnosis of diseases. Specifically, miRNAs, which are short RNA molecules consisting of 21–23 nucleotides, play a significant role in the development and progression of tumors. In addition, p53 is a gene that suppresses tumor growth and is altered in over 50% of all cancerous conditions [6]. Urinary miRNA detection has garnered significant interest in comparison with blood and tissue samples, primarily because of the noninvasive and convenient nature of urine collection. Hence, the identification of urine single-stranded nucleic acids can serve as a crucial method for diagnosing and tracking malignancies. Polymerase chain reaction (PCR) technology have been widely employed as a conventional approach for detecting nucleic acids, specifically single-stranded RNA (ssRNA) and single-stranded DNA (ssDNA) due to their exceptional sensitivity and precision [7, 8]. However, PCR-based detection approaches require costly and accurate heat cycling instruments, which restrict their use in situations with limited resources.

Several isothermal amplification techniques have recently been introduced for nucleic acids detection [9–13]. Out of these approaches, rolling circle amplification (RCA) [14–16], catalytic hairpin assembly technology (CHA) [17, 18], hybridization chain reaction (HCR) [19], EXPAR [20], and primer exchange reaction (PER) [21] have been commonly used. Among them, the entropy-driven CHA and HCR arise from the complementary pairing of bases. These amplification methods that do not require enzymes have a comparatively lower effectiveness in amplifying DNA and are affected by significant interference from background signals. Both EXPAR and PER are highly efficient amplification techniques for detecting nucleic acids [22, 23]. The target molecule, which possesses a particular sequence at its 3′-terminal, serves as a primer to stimulate signal amplification. However, many ssRNA and ssDNA molecules have lengthy nucleotide sequences and ambiguous 3′-terminals. As a result, ssRNA and ssDNA cannot be used directly as primers to initiate amplification events. RCA systems utilize rapid, accurate, and isothermal amplification procedures, requiring no specialized equipment, in contrast to the aforementioned technologies [16, 24, 25]. Nevertheless, the biosensors based on RCA exhibit insufficient sensitivity and are plagued by the inevitable issue of nonspecific amplification [14, 15, 26]. Therefore, it remains a highly difficult endeavor to develop precise and efficient methods for measuring ssRNA and ssDNA.

We develop in this study a new assay by utilizing handgrip-ring structure sensing probe assisted multiple signal amplification strategy for the rapid and ultrasensitive detection of target single-stranded nucleic acids. The handgrip-ring structure sensing probe is composed of a circle ring and a hairpin structure probe, which is responsible for the integrating the specific target recognition, RCA, and self-priming-assisted signal recycling. Based on this, a large number of nucleic acid G-quadruplexes are generated, which are particularly detected by a commercial fluorescent dye without the need for labeling. The suggested biosensor demonstrated exceptional sensitivity, enabling the measurement of single-stranded nucleic acids sequences with a high sensitivity, even in urine samples.

## 2. Experimental Section

### 2.1. Materials and Reagents

The oligonucleotide sequences for target recognition and signal amplification were obtained from Shanghai Sangon Biotech Co. Ltd. (Shanghai, China). The details of the sequences used in this research are shown in Table S1. T4 DNA ligase, phi29 DNA polymerase, Nb.BbvCI, and thioflavin T (ThT) were provided by Sigma-Aldrich (Shanghai, China). Deoxyribonucleoside 5′-triphosphate mixture (dNTPs) was obtained from New England Biolabs (NEB, UK).

### 2.2. Construction of the Sensing Probe

*Assembly of the H probe*: 2 *μ*L of the H probe (500 nM) was added to a tube containing 22 *μ*L DEPC water. The mixture was heated to 90°C for 10 min and gradually cooled to room temperature.

### 2.3. Detection Procedures of the Established Biosensor

The target with different concentrations (10 *μ*L) was first added to a mixture of handgrip-ring sensing probe (500 nM), which contain 5 *μ*L of 10× Phi29 DNA polymerase buffer (50 mM Tris–HCl at pH 7.5, 10 mM MgCl_2_, 10 mM (NH_4_)_2_SO_4_, and 4 mM DTT) and 25 *μ*L of DEPC water. The obtained mixture was incubated at room temperature for 60 min. Then, 2 *μ*L of phi29 DNA polymerase (1.2 U/L), 2 *μ*L of dNTPs (0.25 mM), 2 *μ*L of ThT (5 *μ*M), and 0.8 U/L of Nb.BbvCI were added into a total volume of 100 *μ*L. The obtained mixture was incubated at 30°C for 120 min. After the reaction was completed, fluorescence signal was detected using a fluorescence spectrometer.

## 3. Results and Discussion

### 3.1. Principle of Single-Stranded Nucleic Acid Detection

**Figure 1:**
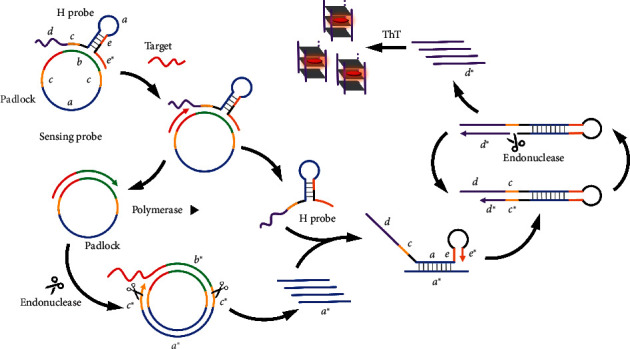
The working mechanism of the proposed method for sensitive and label-free single-stranded nucleic acids detection.

The operational mechanism of the suggested biosensor for detecting single-stranded nucleic acid sequences is illustrated in Figure 1. This approach involves the construction of a sensing probe with a handgrip-ring structure by hybridization between a ring padlock and a hairpin probe. With miRNA as the detection target, we explained the working mechanism of the biosensor. When the target miRNA is present, it precisely attaches to the circular padlock by complementary base pairing. Subsequently, the 3′ end of the target miRNA attached to the circular padlock is consistently elongated by phi29 DNA polymerase at a constant temperature, resulting in the release of the H probe from the handgrip-ring structure sensing probe. During the process of the RCA, the transcribed “*c*^∗^” can be identified and cleaved by the nicking enzyme Nb.BbvCI. Under the assistance of DNA polymerase, “*b*^∗^” is elongated, in which process “*a*^∗^” is displaced from the padlock. As a result, a portion of “*a*^∗^” fragments can be utilized to activate the released H probe by forming a hybrid with the “*a*” segment of the H probe, leading to the unfolding of the hairpin structure of the H probe. According to this, “*e*^∗^” forms a hybrid with the section labeled “*e*” and serves as a primer to promote the elongation of the chain (“*d*^∗^” and “*c*^∗^”). The enzyme Nb.BbvCI specifically identifies and cleaves the DNA sequences containing the “*c*^∗^” fragment, resulting in the creation of a nicking site. By the cooperation of DNA polymerase and endonuclease, a large amount of “*d*^∗^” sequences are produced. G-quadruplexes can be formed by the “*d*^∗^” sequences, and these structures can be precisely detected using a commercially available fluorescent dye called ThT without the need for labeling.

### 3.2. Feasibility of the Method

**Figure 2:**
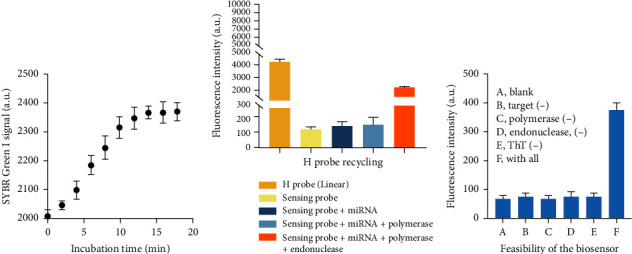
Feasibility of the approach for target sequences detection. (a) SYBR Green I signal of the H probe during the assembly process. (b) Fluorescence intensity of the FAM labeled H probe during the recycling process of H probe. (c) ThT values of the approach for target detection when essential experimental components existed or not. A, blank; B, target (−); C, polymerase (−); D, endonuclease, (−); E, ThT (−); F, with all. Data were expressed as mean ± standard deviations, *n* = 3 technical replicates.

The H probe has a crucial function in directing the succeeding self-priming-mediated signal cycle and enhancing the specificity of the detection approach. Thus, the SYBR Green I dye was employed to confirm the assembly of the H probe. The SYBR Green I probe exhibits specific binding to double-stranded DNA structures and produces a fluorescence signal. As depicted in Figure 2(a), the fluorescence signal of SYBR Green I was significantly low when the H probe was in a linear condition. When the H probe was heated to 90°C and subsequently cooled to room temperature, the SYBR Green I signal exhibited a progressive increase over time, suggesting that the H probe had formed a hairpin structure.

Subsequently, fluorescent dye and quenching groups were attached to both ends of the H probe in order to confirm the stability of released H probe with the sensing probe by chain extension facilitated by the target.  Figure 2(b) demonstrates a notable drop in fluorescence signal when the H probe was constructed in a stem-ring configuration. In the presence of both the target and DNA polymerase, the fluorescence intensity of the H probe remains unchanged, indicating that the H probe is able to maintain its hairpin structure even after being liberated from the sensing probe. Upon the addition of endonuclease to the sensing system, a notable increase in fluorescence signal is detected, suggesting that the “*a*^∗^” sequence formed is responsible for the opening of the H probe.

In order to verify the practicality of our suggested biosensor for detecting single-stranded nucleic acids, we conducted a fluorescent experiment.  Figure 2(c) clearly demonstrates that the fluorescence responses were significantly improved only when all necessary experimental components were included. The absence of each experimental component, such as the phi29 polymerase, nicking enzyme Nb.BbvCI, and ThT, resulted in recorded fluorescence intensities that did not exhibit any noteworthy disparity compared to the control group.

### 3.3. Optimization of the Experimental Condition

**Figure 3:**
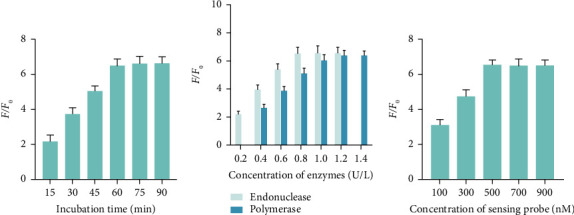
Optimization of experimental parameters. Fluorescence ratio of the approach when detecting target sequences with different incubation time (a), concentration of enzymes (b), and concentration of the sensing probe (c). Data were expressed as mean ± standard deviations, *n* = 3 technical replicates.

In order to optimize the performance of the constructed biosensor, we conducted an investigation into several experimental parameters. The *F*/*F*_0_ (*F*, the fluorescence intensity at 480 nm when target existed and *F*_0_, the fluorescence intensity at 480 nm without target) was used to compare the detection performance of the method. As depicted in Figure 3(a), the fluorescence ratio exhibited a progressive rise from 10 to 60 min as the reaction time increased, eventually reaching a state of equilibrium after 60 min. Based on these findings, the optimal duration for the rapid and accurate identification of the target was determined to be 60 min. Subsequently, we compared the fluorescence signals in order to ascertain the most favorable concentration of both the endonuclease and polymerase. According to the data presented in Figure 3(b), the efficiency of *F*/*F*_0_ steadily rose until reaching its highest point at an endonuclease concentration of 0.8 U/L and a DNA polymerase concentration of 1.2 U/L. After that, the *F*/*F*_0_ declined due to an increase in background fluorescence (*F*_0_). The optimal enzyme concentrations for the endonuclease and DNA polymerase in the subsequent experiments were determined to be 0.8 and 1.2 U/L, respectively. Furthermore, the optimal concentration of the sensing probe was determined. The sensing probe concentration of 500 nM was selected for subsequent studies due to its significantly improved *F*/*F*_0_, as seen in Figure 3(c).

### 3.4. Sensitivity and Specificity of the Proposed Method for miRNA Detection

**Figure 4:**
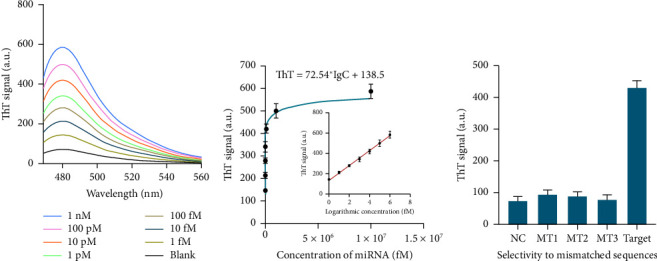
Analytical performance for miRNA detection. (a) Fluorescence spectrum of the approach when detecting different concentrations of miRNAs. (b) Correlation between the calculated ThT signals and the concentration of miRNA. (c) ThT signals of the approach when detecting target miRNA and mismatched sequences. Data were expressed as mean ± standard deviations, *n* = 3 technical replicates.

In order to assess the sensitivity of the strategy, the method was used to identify target miRNA within a concentration range of 1 fM–1 nM. The data shown in Figure 4(a) indicate the peak fluorescence intensities of the approach elevated with the concentration of miRNA increased. A direct correlation between the concentration of the target and the corresponding increase in ThT levels was obtained as ThT = 72.54 ∗ lgC + 138.5, *R*^2^ = 0.9963, when plotting the ThT values against the logarithm of the target concentration. In this linear equation, ThT is the recoded fluorescence intensity and C is the concentration of the target. This relationship was valid within the concentration range of 1 fM–1 nM (Figure 4(b)). The limit of detection (LOD) was determined to be 376 aM, which is equivalent to or even superior to the LODs achieved by earlier isothermal amplification methods.

The specificity of this strategy was evaluated by using different types of target samples, including those with no target DNA, perfectly matched target DNA, and mismatched DNA (with single base mismatch, MT1; with two bases mismatch, MT2; with three bases mismatch, MT3; and with noncomplementary sequence, NC), respectively. According to the data shown in Figure 4(c), the ThT values for the nonspecific target (MT1, MT2, MT3, and NC) were much lower compared to the ThT values for the perfectly matched target miRNA. This experiment proved the efficacy of the biosensor in accurately detecting the target miRNA in different types of samples, while minimizing false positives. The proposed biosensor demonstrated exceptional specificity by effectively distinguishing even a single base mismatched target miRNA from a perfect-matched target miRNA, which can be attributed to the design of the handgrip-ring structure sensing probe. This design effectively minimizes the likelihood of interfering molecules opening the H probe and triggering a nonspecific reaction.

### 3.5. Sensitivity and Specificity of the Proposed Method for p53 Gene Detection

**Figure 5:**
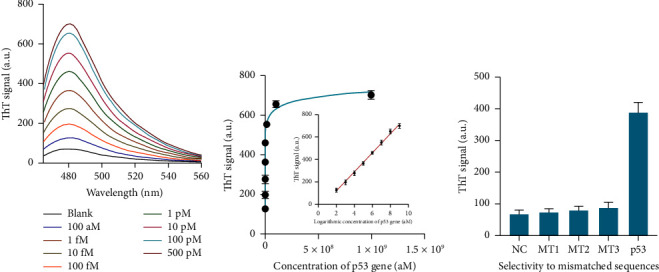
Analytical performance for p53 detection. (a) Fluorescence spectrum of the approach when detecting different concentrations of p53 gene. (b) Correlation between the calculated ThT signals and the concentration of p53 gene. (c) ThT signals of the approach when detecting target sequence and mismatched sequences. Data were expressed as mean ± standard deviations, *n* = 3 technical replicates.

In order to showcase the efficacy of the constructed biosensor in accurately detecting the desired DNA targets, a range of quantities of the p53 gene were individually evaluated in ideal conditions.  Figure 5(a) illustrates a considerable increase in fluorescence intensity as the concentration of the p53 gene increases from 100 aM to 500 pM. The interplay between the sensing system's fluorescence intensity and the logarithm of the p53 gene concentration is shown in Figure 5(b). The calibration curve's regression equation was represented as ThT = 85.86 ∗ lgC − 55.42 (*R*^2^ = 0.9974). The LOD, calculated as three times the standard deviation divided by the slope ((3*σ*/slope)), was determined to be 45.3 aM. The fluorescence changes observed in Figure 5(c) demonstrated a large variation across the interfering sequences, indicating a strong selectivity of our proposed technique for the target p53 gene.

### 3.6. Clinical Application of the Method

**Figure 6:**
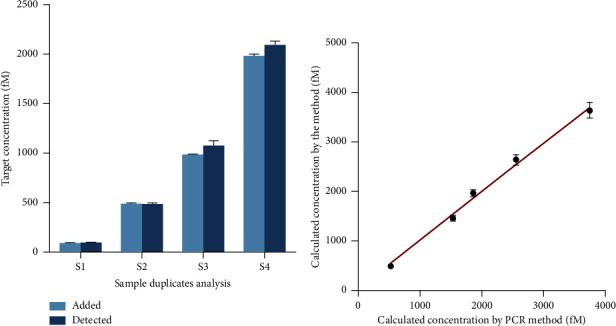
Clinical application of the method. (a) Recovery test for the target detection. (b) Correlation between the calculated target concentration by the method and PCR method. Data were expressed as mean ± standard deviations, *n* = 3 technical replicates.

In recent years, the use of miRNA in urine as a tumor marker has gained increasing attention due to its noninvasive nature and simple collecting character. Several miRNAs indicators in urine have been demonstrated to be linked to the identification of malignancies. To assess the potential clinical use of the developed technique, we diluted the synthetic miRNA in commercial urine to various concentrations. Subsequently, we employed the developed biosensor to measure the miRNA content in the samples.  Figure 6(a) displays miRNA recovery in urine samples of the biosensor exhibiting a range of 98.88%–103.12%, showing a good level of stability. Following that, the presence of miRNAs in the samples was identified using both the developed biosensor and the PCR method. The results demonstrated a strong concordance between the developed method and the PCR method with a high correlation coefficient of 0.9954, thus confirming the approach's potential clinical significance (Figure 6(b)).

## 4. Conclusions

Essentially, a new method for detecting single-stranded nucleic acid sequences has been created. This method relies on a sensing probe with a handgrip-ring construction, which enables multiple signal amplification. The constructed biosensor has several distinct advantages in diagnosis and detection when compared to other methods [27–29]. First, this biosensor uses a handgrip-ring structure sensing probe, which allows for specific target recognition and reduces background signals, which results in highly accurate discrimination and detection of mutations in ssRNA or ssDNA. Second, the sensing platform is equipped with a self-priming strategy that greatly enhances signal amplification efficiency. Lastly, single-stranded nucleic acid sequences detection can be performed in a cost-efficient and label-free mode by utilizing the fluorescence response of G-quadruplexes to ThT. The suggested biosensor has the capability to achieve highly sensitive target identification, even under complex experimental settings, such as urine. We expect that the developed assay will enhance the detection of the target, serving as a novel platform for biological diagnosis.

## Data Availability

The data used to support the findings of this study are available from the corresponding author upon request.

## Supporting Information

Supporting Information Table S1: Oligonucleotide sequences used in this work.
