# Supplementary material for: Handgrip-Ring Structure Sensing Probe Assisted Multiple Signal Amplification Strategy for Sensitive and Label-Free Single-Stranded Nucleic Acid Analysis
Source: J Anal Methods Chem. 2024 Oct 18;2024:6832856. doi: 10.1155/2024/6832856 (PMC11511596; doi:10.1155/2024/6832856)
Supplement: Supporting Information — Table S1: Oligonucleotide sequences used in this work. [file 6832856.f1.docx]

**Supporting information**

**Table S1**. Oligonucleotide sequences used in this work.

| **Name** | **Sequence (5’ to 3’)** |
| --- | --- |
| H probe | ATC CCT ATC CCT ATC CCT ATC CCT CCT CAG CCA AAA ACA CAA CTG GAC GAC TTG AAA AAC TAT ACA ACC TAC TAC CTC A TTT CAA GTC GTC CAG AAG AAA |
| p53 fragment | Ttt gag gtg cgt gtt tgt gcc tgt cct ggg a |
| Padlock-ssRNA | P-TAA CTC TCG TCG AAA AAG TGT GTT TCT TTT TAC CTC AGC ATC AAC TAT ACA ACC TAC TAC CTC A ACC TCA GCA AAC CC |
| Target miRNA | AAU CCG UCG ACG AGA GUU AGG GUU |

**Supplemented experimental section**:

*Detection of target in Urine*

The practicability of the proposed method for p53 gene detection in urine samples was performed using fresh urine spiked with the target. The urine was collected from three healthy volunteers. Then, different amount of p53 gene was directly added to the 10-fold dilution of urine samples. The detection was similar to the above-mentioned approach.
